# A Highly Sensitive Silicon Nanowire Array Field Effect Transistor Biosensor for Detecting HBV-DNA and AFP

**DOI:** 10.3390/s25206385

**Published:** 2025-10-16

**Authors:** Peng Sun, Mingbin Liu, Yongxin Zhang, Chaoran Liu, Xun Yang

**Affiliations:** 1 School of Information Technology, Luoyang Normal University, Luoyang 471934, China; sunpeng1@lynu.edu.cn (P.S.); zyx@lynu.edu.cn (Y.Z.); 2 School of Electronic and Information Engineering, China West Normal University, Nanchong 637002, China; liumb926@163.com; 3 Ministry of Education Engineering Research Center of Smart Microsensors and Microsystems, College of Elecctronics and Information, Hangzhou Dianzi University, Hangzhou 310018, China; liucr@hdu.edu.cn

**Keywords:** SiNW-array FET, highly sensitive, joint detection, hepatitis B virus

## Abstract

Chronic hepatitis B poses a serious threat to human health and life, and early diagnosis is essential to improving patient cure rates. Hepatitis B virus (HBV) and Alpha-fetoprotein (AFP) are two key biomarkers for diagnosing chronic hepatitis B. In this study, we propose a silicon nanowire array field effect transistor (SiNW-array FET) biosensor that enables highly sensitive, real-time, and low-cost joint detection of both HBV and AFP. The SiNW-array FET is fabricated using traditional micro-nano fabrication techniques such as self-limiting oxidation and anisotropic etching, and its morphology and electrical properties were tested. The results show that the diameters of the fabricated silicon nanowires (SiNWs) are uniform and the SiNW-array FET exhibits a strong output signal and high signal-to-noise ratio. Through specific chemical modification on the surface of SiNWs, the SiNW-array FET is highly sensitive and specific to HBV-DNA fragments and AFP, with ultralow detection limits of 0.1 fM (HBV-DNA) and 0.1 fg/mL (AFP). The detection curve of the SiNW-array FET exhibits good linearity within the HBV-DNA concentration range of 0.1 fM to 100 pM and AFP concentration range of 0.1 fg/mL to 1000 pg/mL. More importantly, the device could also detect HBV-DNA successfully in serum samples, laying a solid foundation for the highly sensitive clinical detection of chronic hepatitis B.

## 1. Introduction

Hepatitis B virus (HBV) infection poses a serious threat to human health and life. HBV infection is the main factor leading to the progression of chronic hepatitis B patients’ conditions [1,2]. The risk of chronic hepatitis B patients developing liver cirrhosis, primary liver cancer, and other end-stage liver diseases is 25~40% [3]. It is reported that approximately 240 million people worldwide have a chronic hepatitis B infection. The number of deaths from liver cirrhosis, primary liver cancer, and liver failure caused by HBV infection is about 1 million per year [4]. Therefore, the high-sensitivity detection of HBV virus is of great importance for the diagnosis and treatment of hepatitis B patients.

Because of its simple sampling, minimal invasiveness, and low cost, disease marker detection has been widely adopted for disease screening, diagnosis, and prognostic evaluation, markedly enhancing the feasibility of early detection [5,6,7]. In patients with chronic hepatitis B, not only is the concentration of HBV virus high, but there is also an increase in the concentration of the disease marker Alpha-fetoprotein (AFP) [8]. AFP is a glycoprotein produced and released from injured liver tissue or other tissues. Numerous studies have shown that the concentration of AFP is a significant reference value for the clinical early diagnosis of chronic hepatitis B, liver cirrhosis, and liver cancer [9,10]. Detection of disease markers can provide supplementary information to improve diagnostic accuracy [11], thereby enabling hepatitis B patients to receive timely treatment and increase their survival rates. In summary, the development of joint detection technology that is highly sensitive and selective of HBV-DNA and AFP is of great significance.

Biosensor technology [12,13], with its excellent specificity and sensitivity, has unique advantages in DNA detection. Currently, many methods for DNA detection have been proposed, such as electrochemical impedance testing [14], surface plasmon resonance technology [15], and digital PCR technology [16]. However, these methods still have certain limitations, and precise reading of DNA signals remains a challenge. Achieving high-sensitivity detection still requires expensive and complex equipment. The general clinical detection methods for AFP include chemiluminescence enzyme immunoassay [17] and electrochemical immunoassay [18]. However, these methods have disadvantages such as the need for labeling, long detection time, low sensitivity, and expensive equipment.

Nanomaterials with excellent electrical conductivity offer new solutions to these problems. In particular, silicon nanowire (SiNW), as a highly promising nanomaterial, exhibits superior properties in the fields of optics, electronics, thermodynamics, and mechanics [19,20,21,22]. With its large specific surface area and good biocompatibility, SiNW has wide applications in biomedicine [23,24], disease diagnosis [25,26,27], environmental monitoring [28], and other fields. SiNW-based MEMS sensors [29], due to their larger specific surface area, have high sensitivity. Even when only a few target molecules carrying charges are bound to the surface of SiNW, its electrical conductivity will change significantly. Based on this significant characteristic, the silicon nanowire field effect transistor (SiNW-FET) shows a higher sensitivity and specificity in detecting specific target molecules and can be used to detect target molecules such as DNA, proteins, and cells [30,31,32,33,34,35]. The key to achieving high-selectivity and high-sensitivity detection of biomolecules is to fabricate SiNW array biosensors with high yield and low cost. In the commonly used SiNW-FET sensor, the number of SiNWs is usually only a few or a dozen [36,37]. The size and shape of the SiNWs prepared by chemical vapor deposition are difficult to control and their arrangement is non-uniform. The top-down method for fabricating SiNWs has solved the drawbacks of chemical vapor deposition, but this technique requires expensive lithographic equipment [38,39]. During the detection of biomolecules, the signals generated by the binding of probe molecules and target molecules are relatively weak, and SiNWs are unable to detect the changes of the target molecules on the surface.

To address the above issues, we have fabricated a SiNW-array FET device using traditional micro-nano fabrication techniques such as self-limited oxidation and anisotropic etching, and have performed morphological characterization and electrical property testing on the devices. The test results show that the prepared SiNWs have a uniform diameter and the sensor exhibits excellent signal strength and signal-to-noise ratio, achieving highly sensitive and specific detection of specific DNA fragments of the hepatitis B virus and the disease marker AFP. The detection sensitivity can reach 0.1 fM (HBV-DNA) and 0.1 fg/mL (AFP), respectively. Good degree of linearity value of the device is found for the detection curves in the HBV-DNA concentration range of 0.1 fM to 100 pM and AFP concentration range of 0.1 fg/mL to 1000 pg/mL. In summary, the SiNW-array FET can achieve the highly sensitive and accurate detection of two analytes, greatly improving the detection efficiency.

## 2. Materials and Methods

### 2.1. The Fabrication Process of SiNW-Array FET

**Figure 1:**
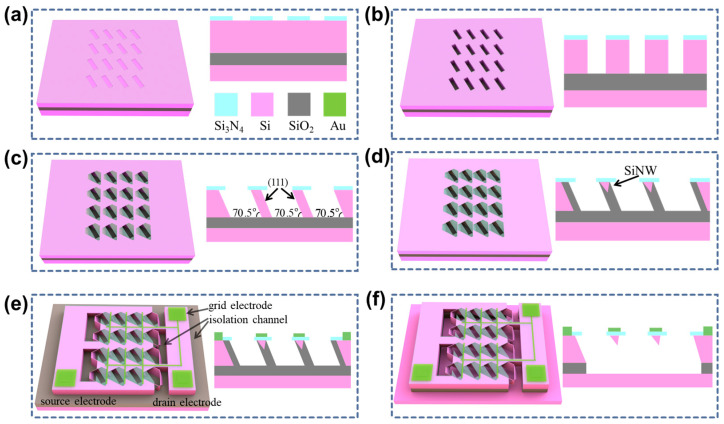
The fabrication process of the SiNW-array FET. (**a**) The window array was formed by photolithography and reactive ion etching; the image on the right is the cross-sectional view of the device. (**b**) The etching grooves were formed by deep reactive ion etching. (**c**) The tilted silicon walls were created by anisotropic wet etching. (**d**) The SiNW-array was formed by self-limiting oxidation. (**e**) The fabrication of electrodes and isolation channels. (**f**) The oxide layer was removed to expose the SiNWs.

To address the challenge of mass production of SiNWs, in our earlier work, we have used (111)-oriented Silicon-On-Insulator (SOI) wafer and employed traditional microfabrication processes to fabricate wafer-scale, highly controllable, and uniformly sized SiNW-array FET [22,40]. As shown in Figure 1, the entire manufacturing process of the SiNW-array FET is compatible with CMOS technology, including processes such as reactive ion etching, photolithography, and wet etching. The fabricated SiNWs have an inverted triangular array structure, and the SiNW-array FET exhibits a higher signal-to-noise ratio and signal strength. The novel gate structure effectively prevents the fracture of SiNWs. Compared with a single SiNW, the output signals from each SiNW in the array are superimposed, enabling a larger output current. The production cost of the single SiNW-array FET is about USD 0.7023.

### 2.2. Materials and Reagents

Glutaraldehyde (GA), ethanolamine, and absolute ethanol were purchased from Shanghai Macklin Biochemical Co., Ltd. (Shanghai, China). 3-aminopropyltriethoxysilane (APTES) was purchased from Sigma-Aldrich (St. Louis, MO, USA). N-hydroxysuccinimide (NHS) was purchased from Shanghai Guchen Biotechnology Co., Ltd. (Shanghai, China). 1-ethyl-3-(3-dimethylaminopropyl) carbodiimide (EDC) was bought from Shanghai Yuanye Bio-Technology Co., Ltd. (Shanghai, China). AFP and AFP antibodies were bought from Abcam (Cambridge, England). SCCA and APT were purchased from Aladdin Company (Shanghai, China). PBS buffer solution was prepared at Shanghai Little Turtle Technology Co., Ltd. (Shanghai, China). The DNA sequences used in the experiment were synthesized by Shanghai Generay Biotech Co., Ltd. (Shanghai, China). In this experiment, the detection target is a special DNA sequence of the hepatitis B virus (5′-AAGAACCAACAAGAAGATGAGGCATA-3′). The DNA probe molecule is complementary to this target DNA sequence and is modified with a carboxyl group at the 5′ end (5′-COOH-CCCCCCTATGCCTCATCTTCTTGTTGGTTCTT-3′). According to the National Center for Biotechnology Information (NCBI), the nucleotide sequence of the HBV genome is numbered NC_003977, and the probe sequence corresponds to nucleotides 422–447 of NC_003977. The sequences of probe DNA, HBV-DNA, and non-complementary pairing DNA with probe DNA are listed in Table 1.

### 2.3. Surface Modification Process of the SiNW-Array FET

**Figure 2:**
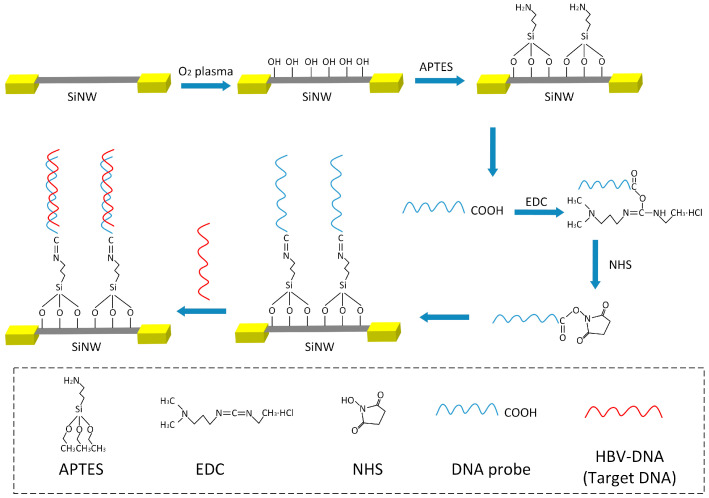
The process of HBV-DNA probe modification and detection of target HBV-DNA on the surface of SiNWs.

In this design, the surface probe HBV-DNA and AFP antibody of SiNWs are modified by chemical bond combination. The modification process of the probe HBV-DNA is shown in Figure 2. First, the cleaned and dried SiNW-array FET was placed into an oxygen plasma machine, with the power set at 120 W for 5 min, and the surface of the SiNWs were modified with hydroxyl groups. Then, the SiNW-array FET was immersed in a 2% APTES ethanol solution overnight for silanization treatment, the surface of the SiNWs were modified with amino groups. After that, a mixture of 5 μM DNA probe solution, 0.4 mM EDC solution, 0.1 mM NHS solution, and standard PBS solution, in a volume ratio of 4:5:5:6, was evenly mixed and dropped onto the surface of SiNWs for incubation, and left undisturbed for 2 h. Here, EDC solution and NHS solution can be used to activate carboxyl groups, promoting the dehydration reaction between carboxyl and amino groups, thereby increasing the coupling rate of DNA probes on the surface of SiNWs. When HBV-DNA solution is dropped onto the surface of SiNWs, the binding of probe DNA and HBV-DNA will cause changes in the electrical conductivity of SiNWs. Due to the relative stability of DNA molecules, the storage time of HBV-DNA sensor based on SiNW-array FET at room temperature is approximately one week.

**Figure 3:**
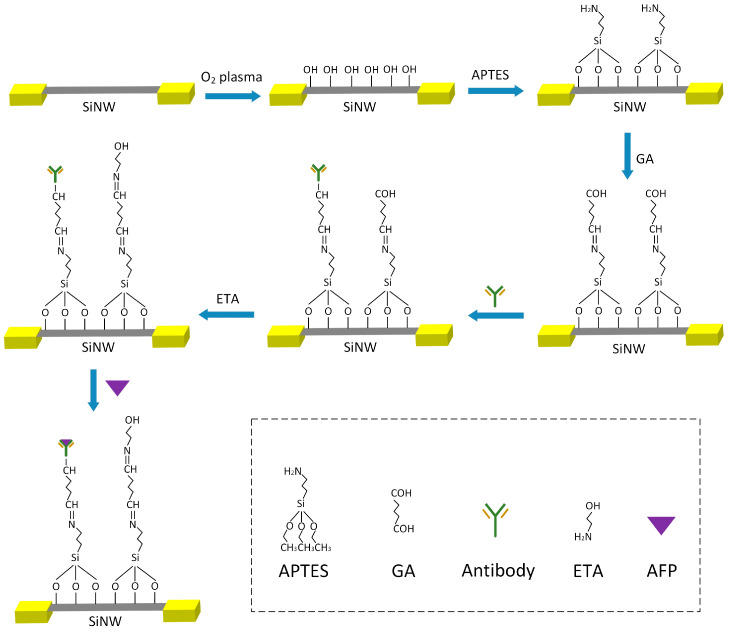
The process of antigen modification and AFP detection on the surface of SiNWs.

The modification process of the AFP antibody is shown in Figure 3. After the SiNW-array FET was modified by silanization, 10% GA solution was dropped onto the surface of SiNWs and left undisturbed for 2 h. The chemical reaction between aldehyde groups and amino groups can modify GA onto its surface. Then, the AFP antibody solution was added to couple it to the surface of SiNWs. After that, the ETA solution was dropped to combine with the unreacted aldehyde groups, reducing the interference current caused by external interfering substances binding to the unreacted aldehyde groups. When the AFP solution is added and detected, the binding of the AFP antibody and AFP will cause changes in the electrical conductivity of SiNWs. Due to the easy deformation and inactivation of antibodies, the storage time of an AFP sensor based on the SiNW-array FET at room temperature is approximately three days.

### 2.4. Experimental Conditions

In this experiment, we used a Keithley 2450 digital source meter to test the electrical parameters of the sensing device. As the voltage was applied between the source and drain of the sensing device by using the Keithley 2450 (Keithley Instruments, Solon, OH, USA), the current between the source and drain could be measured in real time.

## 3. Results and Discussion

### 3.1. Morphology Characterization and Electrical Properties of SiNW-Array FET

**Figure 4:**
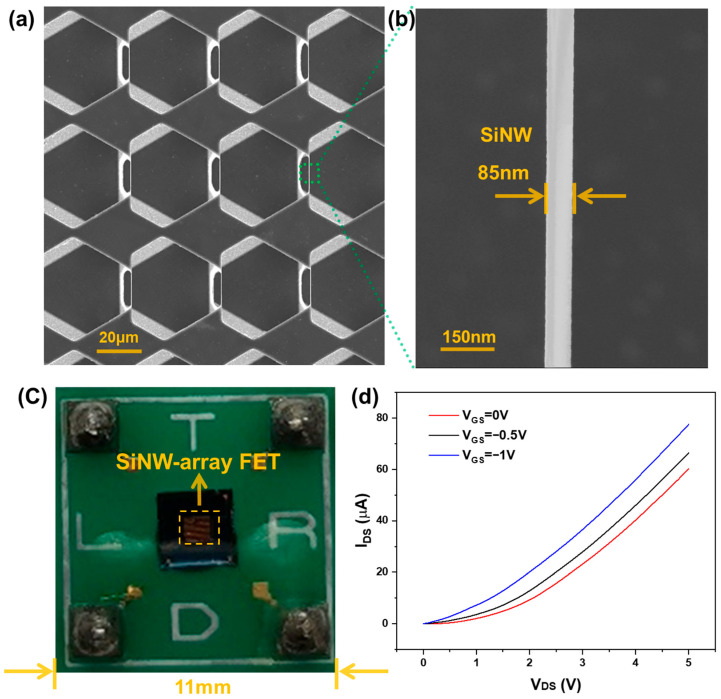
(**a**) The electron microscopy image of the suspended SiNW-array. (**b**) The localized amplification of a single SiNW. (**c**) Physical image of SiNW-array FET. (**d**) Electrical output characteristic curve of SiNW-array FET.

It is important to characterize the external morphology of the fabricated SiNW-array FET. Figure 4a presents the electron microscopy image of the suspended SiNW-array. These uniformly sized SiNWs work together in parallel to achieve the superposition of detection signals. Figure 4b shows a magnified view of a single SiNW, revealing its morphology with a diameter of approximately 85 nm.

Figure 4c shows the physical image of the SiNW-array FET after a series of packaging steps, including die bonding and encapsulation. The central area of the image is the SiNW-array FET, with source, drain, and gate pins at the lower left, lower right, and upper right corners, respectively. After surface-specific chemical modification, the packaged SiNW-array FET is fabricated into a specific biosensor for specific detection of target molecules.

After the SiNW-array FET is packaged, its electrical properties need to be tested. The fabricated SiNW-array FET exhibits the transfer characteristics of a P-channel depletion MOSFET. When $V_{GS}=0\;V$, the conductive channel has already been formed. As $V_{GS}$ decreases, the increase in the concentration of holes in the channel leads to an increase in the electrical conductivity of the silicon nanowire. Under the same $V_{DS}$ conditions, the output current increases. Figure 4d shows the output characteristic curves of the SiNW-array FET at different gate voltages. From the figure, it can be seen that when $V_{DS}$ is constant, as $V_{GS}$ decreases, the output current $I_{DS}$ increases, which is consistent with the transfer characteristics of a P-channel depletion MOSFET. Therefore, it can be inferred that when the SiNW-array FET is used as a sensor and when the target molecules with the charges are absorbed on the surface of the SiNW-array FET, its gate voltage is changed, leading to the change in the output current. This is the most critical point for the SiNW-array FET as a biosensor.

### 3.2. The Sensing Mechanism of the SiNW-Array FET

**Figure 5:**
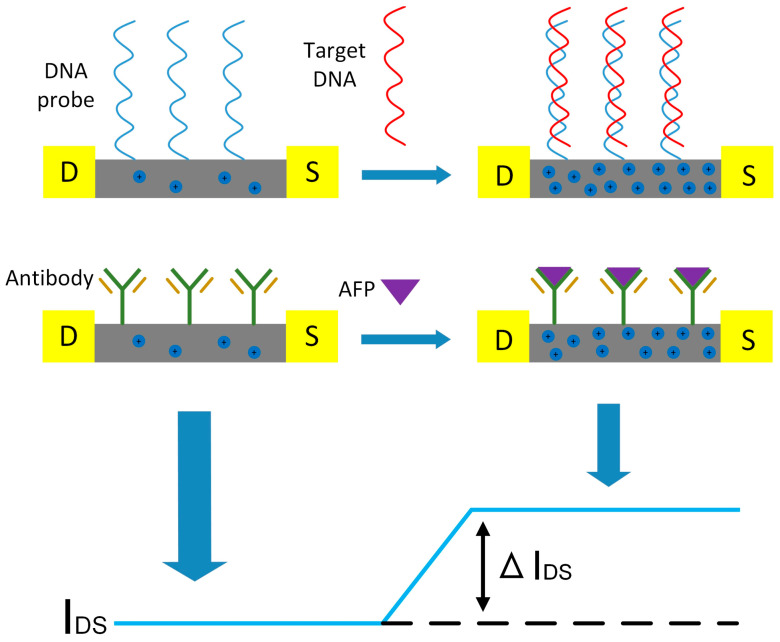
Sensing mechanism of the p-type SiNW-FET sensor for detecting HBV-DNA and AFP molecules.

When SiNW-FET is employed for biomolecular detection, bio-probe molecules are typically immobilized on the surface of the SiNWs. The binding of target molecules carrying charges (such as DNA, proteins, or antigens) to the probe molecules induces either depletion or accumulation of charge carriers in the conduction channel, thereby modulating the conductivity of the SiNW-FET. This effect arises because the charged target molecules effectively introduce an electric field at the gate region of the FET device. The fabricated sensor device is a p-type SiNW-array FET. Owing to the larger specific surface area of the SiNWs and the fact that each individual nanowire can function as a sensing unit, the device exhibits high sensitivity to changes in the external electric field environment. During the detection process, HBV-DNA probe molecules or AFP antibody molecules are functionalized on the surface of the SiNWs. When these probe molecules bind to target molecules, the negative charge carried by the target molecules induces a negative electric field at the gate surface of the SiNW-FET. This electric field increases the hole concentration in the conduction channel of the FET, thereby enhancing the conductivity and leading to an increase in the output current. Figure 5 illustrates the sensing mechanism of the p-type SiNW-FET sensor for detecting HBV-DNA and AFP molecules.

### 3.3. Sensitivity of the SiNW-Array FET

Sensitivity is a very important indicator of SiNW sensors and reflects the detection capability of the sensor to a certain extent. The cross-section of the SiNW prepared in this paper is triangular in shape. Figure S1 shows the schematic diagram of the SiNW model. The initial current of the SiNW device under a constant source-drain voltage is defined as $I_{D,0}$, and the current change on the surface of the SiNW caused by biomolecules is defined as ${\Delta I}_{D}$. The sensitivity of the device can be expressed as

(1) $$\frac{{\Delta I}_{D}}{I_{D,0}}=\frac{I_{D}{\;-\;I}_{D,0}}{I_{D,0}}$$

The SiNW-array prepared in this paper has a uniform diameter, with sizes of around 85 nm. It can be inferred that the sensitivity of each individual SiNW will exhibit good uniformity. During operation, the SiNWs within the array achieve the superposition of output signals, resulting in a larger and more stable total output current. Most SiNWs reported in the literature currently have current signal outputs at the nanoampere level [37], which necessitates the use of relatively expensive and sophisticated equipment for sensing signal detection. The signal strength of the SiNW devices fabricated in this paper can reach the microampere level, representing a significant enhancement in signal intensity. Moreover, compared to SiNW-FET, the signal-to-noise ratio of the SiNW-array FET is also greatly improved. The signal-to-noise ratio of the SiNW-array sensor can be expressed as

(2) $$SNR=\frac{NS}{m}$$

Here, SNR represents the signal-to-noise ratio of the sensor, N represents the number of SiNW, S represents the output signal strength of a single SiNW, and m represents the random noise in the sensor caused by the external environment. It can be concluded that the SiNW-array not only enhances SNR of the device, but also simplifies the detection circuit, thereby facilitating the application of SiNW devices.

**Figure 6:**
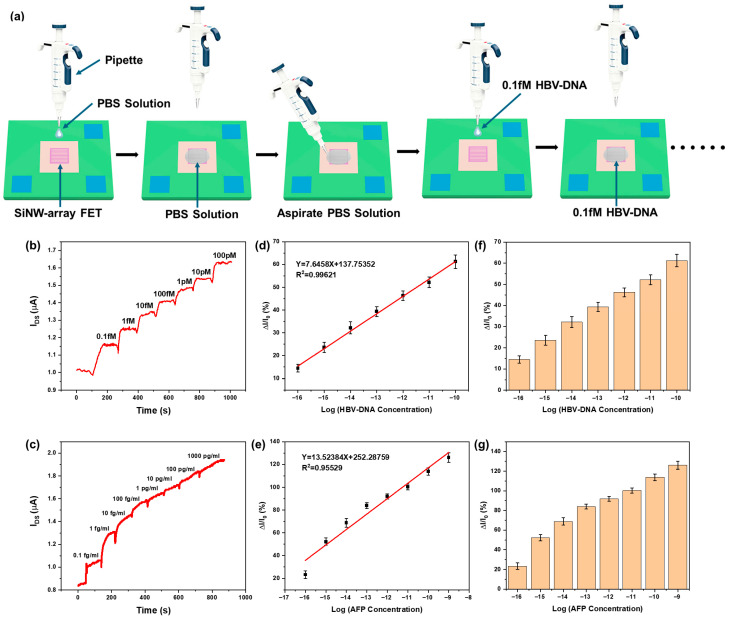
(**a**) Testing process image of HBV-DNA. (**b**) Current response of the SiNW-array FET at distinct target HBV-DNA solution concentrations. (**c**) Current response of the SiNW-array FET at distinct AFP solution concentrations. (**d**) Normalized current fitting curves of the SiNW-array FET at distinct HBV-DNA solution concentrations. (**e**) Normalized current fitting curves of the SiNW-array FET at distinct AFP solution concentrations. (**f**) Histogram of the relative current change of the SiNW-array FET at distinct target HBV-DNA solution concentrations. (**g**) Histogram of the relative current change of the SiNW-array FET at distinct AFP solution concentrations.

After modifying the surface of the SiNW-array FET with probe molecules, real-time detection of target molecule solutions at different concentrations was performed. In the experiment, we added PBS solution to the high-concentration target molecule solutions for continuous dilution, producing a series of HBV-DNA and AFP solutions of different concentrations. The pH value of the PBS solution (137 mM NaCl, 2.7 mM KCl, 10 mM Na_2_HPO_4_, and 2 mM KH_2_PO_4_) is approximately 7.4. As shown in Figure 6a, taking HBV-DNA detection as an example, we first used a pipette to drop a volume of 6 µL PBS solution onto the surface of SiNWs that have been modified with probe DNA. When the current between the source and drain stabilized, it was used as the reference current $I_{0}$. The magnitude of $I_{0}$ was approximately 1 mA, while the voltage between the source and drain was about 200 mV. Then, we used a pipette to aspirate the PBS solution that was previously dripped onto the surface of the SiNWs. Then we quickly used the pipette to drip the HBV-DNA solution with a concentration of 0.1 fM onto the surface of the SiNWs, aspirate the HBV-DNA solution after the current stabilized and then drip the HBV-DNA solution with a concentration of 1 fM onto the surface of the SiNWs. After the current stabilized, we aspirated the HBV-DNA solution that was dripped again. Following the above steps can sequentially detect HBV-DNA solutions with concentrations of 10 fM, 100 fM, 1 pM, 10 pM, and 100 pM. The process of detecting AFP using SiNWs is consistent with the process of detecting HBV-DNA. Figure 6b,c represents the current response curve of the SiNW-array FET to different concentrations of HBV-DNA (0.1 fM to 100 pM) and AFP (0.1 fg/mL to 1000 pg/mL), respectively. The output current $I_{DS}$ gradually increases when the concentration of HBV-DNA and AFP solution increases. For the SiNW-array FET, within a certain concentration range, the higher the concentration of target molecules in the solution, the more target molecules are adsorbed on the surface of the silicon nanowire, resulting in a higher current signal. Figures S2 and S3 illustrate the binding process between probe molecules and target molecules. Figure 6d,e shows the normalized current fitting curves of the SiNW-array FET at distinct DNA and AFP concentrations, respectively, both of which exhibit good linearity. Current change rates of the sensor device under DNA concentrations of 0.1 fM, 1 fM, 10 fM, 100 fM, 1 pM, 10 pM, and 100 pM are 14.52%, 23.62%, 32.17%, 39.34%, 46.27%, 52.15%, and 61.22%, respectively. Current change rates of the sensor device under AFP concentrations of 0.1 fg/mL, 1 fg/mL, 10 fg/mL, 100 fg/mL, 1 pg/mL, 10 pg/mL, 100 pg/mL, and 1000 pg/mL are 23.3%, 52.22%, 68.89%, 83.97%, 91.93%, 100.3%, 113.8%, and 126.06%, respectively.

The limit of detection (LOD) is calculated using the formula $Y_{LOD}=Y_{blank}+3\delta$, in which $Y_{LOD}$ is the threshold current, $Y_{blank}$ is the response of the blank measurement, and δ is the standard deviation of $Y_{blank}$. In this experiment, the value of $Y_{blank}$ is the value of average initial current after adding PBS buffer solution. According to Figure 6b,c, the $Y_{blank}$ values for HBV-DNA and AFP are 1.011 × 10^−6^ A and 8.555 × 10^−7^ A, respectively, while the values of δ for HBV-DNA and AFP are 4.927 × 10^−9^ A and 3.285 × 10^−9^ A, respectively. Therefore, the threshold currents for HBV-DNA and AFP are 1.026 × 10^−6^ A and 8.654 × 10^−7^, respectively. The stable response currents for 0.1 fM HBV-DNA and 0.1 fg/mL AFP are 1.158 × 10^−6^ A and 1.049 × 10^−6^ A, respectively, both of which are higher than their respective threshold currents. The test results indicate that the fabricated SiNW-array FET sensor exhibits excellent sensitivity towards HBV-DNA and AFP. Figure 6f,g shows the histograms of the relative current change of the SiNW-array FET at distinct DNA and AFP concentrations, respectively.

### 3.4. Detection Specificity of the SiNW-Array FET

**Figure 7:**
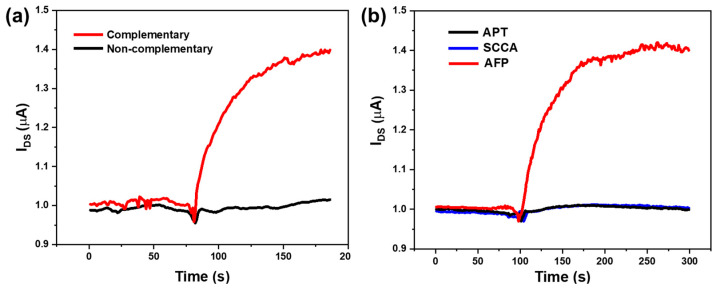
(**a**) Current response of the SiNW-array FET to complementary DNA (HBV-DNA) and non-complementary DNA. (**b**) Current response of the SiNW-array FET to AFP, APT, and SCCA.

To verify the specificity of the SiNW-array FET sensor for HBV-DNA detection, after modifying the surface of the SiNWs with probe DNA molecules, PBS solution was dropped onto the surface of the device. After the current stabilized, non-complementary DNA solution and HBV-DNA solution of the same concentration (100 pM) were successively dropped onto the surface of the device. To facilitate comparison, the response currents corresponding to the two types of DNA solutions are compared in the same figure. The output current curve of the SiNW-array FET sensor is displayed in Figure 7a. It can be shown that the device exhibited good specificity. The non-complementary DNA nearly did not cause change in the output current, while the HBV-DNA caused evident change in the output current. This indicates that the SiNW-array FET modified with probe DNA molecules has good specificity for detecting the specific DNA fragment of the hepatitis B virus.

To verify the specificity of the sensor for AFP detection, Abnormal Prothrombin (APT) and Squamous Cell Carcinoma Antigen (SCCA) were used as interfering substances for AFP. After the surface of the SiNW was modified with AFP antibody molecules, 1 µg/mL APT, 1 µg/mL SCCA, and 1 fg/mL AFP were added sequentially. The output current curve of the SiNW-array FET sensor is displayed in Figure 7b. It can be seen that when APT and SCCA solutions were added, the change in the output current of the device was almost negligible. However, when the AFP solution was added, the current rapidly increased. This indicates that the SiNW-array FET modified with AFP antibody molecules has good specificity for AFP.

### 3.5. Simulation Analysis of Human Serum Samples

**Figure 8:**
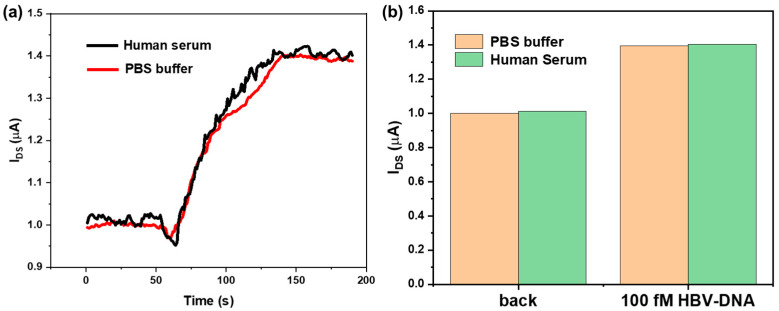
(**a**) Real-time current response of SiNW-array FET to 100 fM HBV-DNA diluted with (black line) or without (red line) human serum. (**b**) Histogram of the current of SiNW-array FET for 100 fM HBV-DNA diluted with (green area) or without (yellow area) human serum.

We used PBS buffer solution and healthy human serum as controls to explore the clinical detection capability of the SiNW-array FET. The human serum samples were obtained from Shanghai Little Turtle Technology Co., Ltd. (Shanghai, China). HBV-DNA was added separately to PBS buffer and healthy human serum to prepare two HBV-DNA solutions with concentrations of 100 fM each. As shown in Figure 8a,b, after dropping the two solutions onto the surface of the SiNW-array FET, two increasing currents with largely similar changes were observed. The above experimental results show the clinical detection capability of the SiNW-array FET.

Table 2 and Table 3 show the detection capabilities of different sensing technologies for HBV DNA and AFP. Compared with most detection methods, the SiNW-array FET has lower LOD and wider linear range for HBV-DNA and AFP. Although some sensors also have good detection capabilities, the manufacturing process of the devices is complex and the detection process is tedious. The SiNW-array FET has advantages such as compatibility with CMOS, high sensitivity, real-time detection, being label free, and low cost.

## 4. Conclusions

In this study, we fabricated the SiNW-array FET using traditional micro-nano fabrication techniques and characterized the morphology of the device. The results showed that the SiNW-array prepared by self-limiting oxidation and anisotropic etching techniques has uniform dimensions, with the size of individual SiNWs being approximately 85 nm. We also tested the electrical characteristics of the SiNW-array FET device, and the test curves indicated that the device exhibited good gate-voltage control characteristics. The SiNW-array FET sensor was employed to achieve highly sensitive and specific detection of specific gene fragments of HBV-DNA and AFP molecules. The SiNW-array FET sensor exhibited good linearity in the detection curves when tested with distinct concentrations of HBV-DNA and AFP solutions, within the DNA concentration range of 0.1 fM to 100 pM and the AFP concentration range of 0.1 fg/mL to 1000 pg/mL. The experimental results also demonstrated that the sensor has good specificity for detecting the specific gene fragments of HBV-DNA and AFP molecules. Meanwhile, in serum samples, the device could also successfully detect HBV-DNA. The SiNW-array FET for detecting HBV-DNA and AFP lays a solid foundation for the highly sensitive clinical detection of chronic hepatitis B.

## Figures and Tables

**Table 1:** Sequences of HBV-DNA probe, HBV-DNA and non-complementary DNA.

DNA	Sequences
HBV-DNA probe	5′-COOH-CCCCCCTATGCCTCATCTTCTTGTTGGTTCTT-3′
complementary DNA (HBV-DNA)	5′-AAGAACCAACAAGAAGATGAGGCATA-3′
non-complementary DNA	5′-CCACTACGCTTCCGCTTACCTCTCAT-3′

**Table 2:** Detection linear range and LOD of Different Sensors for HBV-DNA.

Classification	Linear Range	LOD	Ref.
Electrochemical DNA sensor	1 fM to 10 nM	0.36 fM	[41]
Label-free fluorescent sensor	3 fM to 800 pM	1.6 fM	[42]
Electrochemical biosensor	10 fM to 100 pM	2.5 fM	[43]
DNA hybridization biosensor	10 nM to 3 μM	3.8 nM	[44]
Dual-mode biosensor	100 fM to 100 nM	5.62 fM	[45]
SiNW-array FET in this work	0.1 fM to 100 pM	0.1 fM	This work

**Table 3:** Detection linear range and LOD of Different Sensors for AFP.

Classification	Linear Range	LOD	Ref.
Two-size enzyme-linked immunosensor	20 to 600 ng/mL	9.7 ng/mL	[46]
Two-site ELISA sensor	6 to 100 ng/mL	2 ng/mL	[47]
Electrochemical aptasensor	1 ng/mL to 10 μg/mL	0.3013 ng/mL	[48]
SERRS-based lateral flow immunoassay	10 pg/mL to 500 ng/mL	9.2 pg/mL	[49]
Electrochemical immunosensor	100 fg/mL to 200 ng/mL	18.6 fg/mL	[50]
SiNW-array FET in this work	0.1 fg/mL to 1000 pg/mL	0.1 fg/mL	This work

## Data Availability

All datasets presented in this study are included in the article.

## Supplementary Materials

The following supporting information can be downloaded at: https://www.mdpi.com/article/10.3390/s25206385/s1, Figure S1: The schematic diagram of the SiNW model. Figure S2: The binding process between probe DNA molecules and HBV-DNA molecules on SiNWs. Figure S3: The binding process between antibodies and AFP molecules on SiNWs.
