# Supplementary material for: A Highly Sensitive Silicon Nanowire Array Field Effect Transistor Biosensor for Detecting HBV-DNA and AFP
Source: Sensors (Basel). 2025 Oct 16;25(20):6385. doi: 10.3390/s25206385 (PMC12567904; doi:10.3390/s25206385)
Supplement: Supplementary file 1 [file sensors-25-06385-s001.zip › sensors-3889892-supplementary.pdf]

Supporting Information

A Highly Sensitive Silicon Nanowire Array Field Effect Transistor Biosensor for Detecting HBV-DNA and AFP

Peng Sun [1], Mingbin Liu [2], Yongxin Zhang [1], Chaoran Liu [3], and Xun Yang [2,*]

[1] School of Information Technology, Luoyang Normal University, Luoyang 471934, China

[2] School of Electronic and Information Engineering, China West Normal University, Nanchong 637002, China

[3] Ministry of Education Engineering Research Center of Smart Microsensors and Microsystems, College of Elecctronics and Information, Hangzhou Dianzi University, Hangzhou 310018, China

* Correspondence: yangxun@cwnu.edu.cn

Figure S1 shows the schematic diagram of the SiNW model. The cross-section of the SiNW prepared in this paper is triangular in shape, with a lateral dimension of w, a vertical height of h, and a length of L. For the convenience of calculation and more intuitive observation of the effect of SiNW dimensions on sensitivity, it is generally assumed that $w = h$. The sensitivity of the SiNW can be expressed as:

$$\frac{\Delta I_D}{I_{D,0}} = -\frac{2\sqrt{5}\,N_S}{q n_0 w}$$

In the formula, N_S represents the surface charge density of the SiNW, n_0 represents the initial carrier concentration, and q represents the electron charge. From the above expression, it can be concluded that the sensitivity of the SiNW is approximately inversely proportional to the width. This also theoretically verifies the conclusion that the smaller the dimensions of the SiNW, the larger the specific surface area, and the higher the sensitivity.

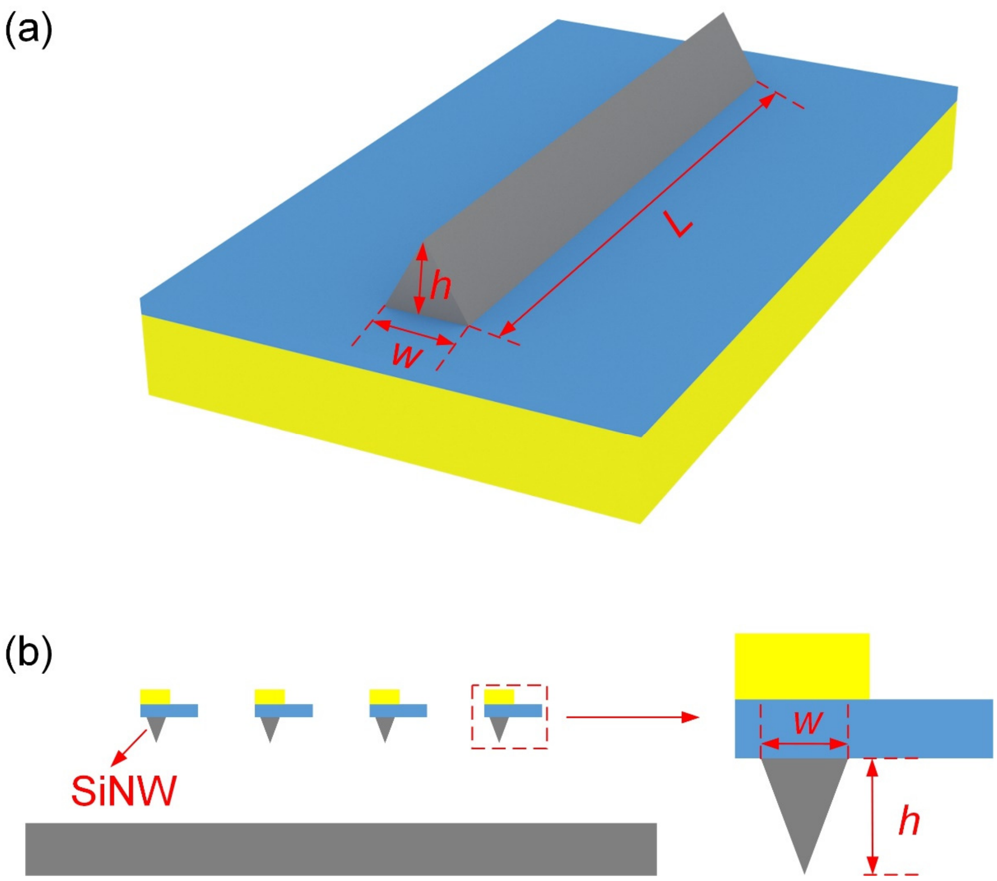

Figure S1. The schematic diagram of the SiNW model. (a) Schematic diagram of silicon nanowire model with triangular cross-section; (b) Schematic diagram of SiNW-array.

As shown in Figure S2, when using the SiNW-array FET to detect HBV-DNA solution with a concentration of 0.1 fM, DNA probe molecules on the SiNWs would bind to HBV-DNA molecules, and the output current of the sensor gradually increased. When a current plateau occurred, it indicated that the DNA probe molecules on the SiNWs no longer bound to HBV-DNA molecules from the solution. When a HBV-DNA solution with a concentration of 1 fM was added, the output current of the sensor could still continue to increase. The reason is that as the concentration of HBV-DNA solution increases, the DNA probe molecules on the SiNWs could continue to bind to HBV-DNA molecules. As shown in Figure S3, the process of detecting AFP is similar to that of HBV-DNA.

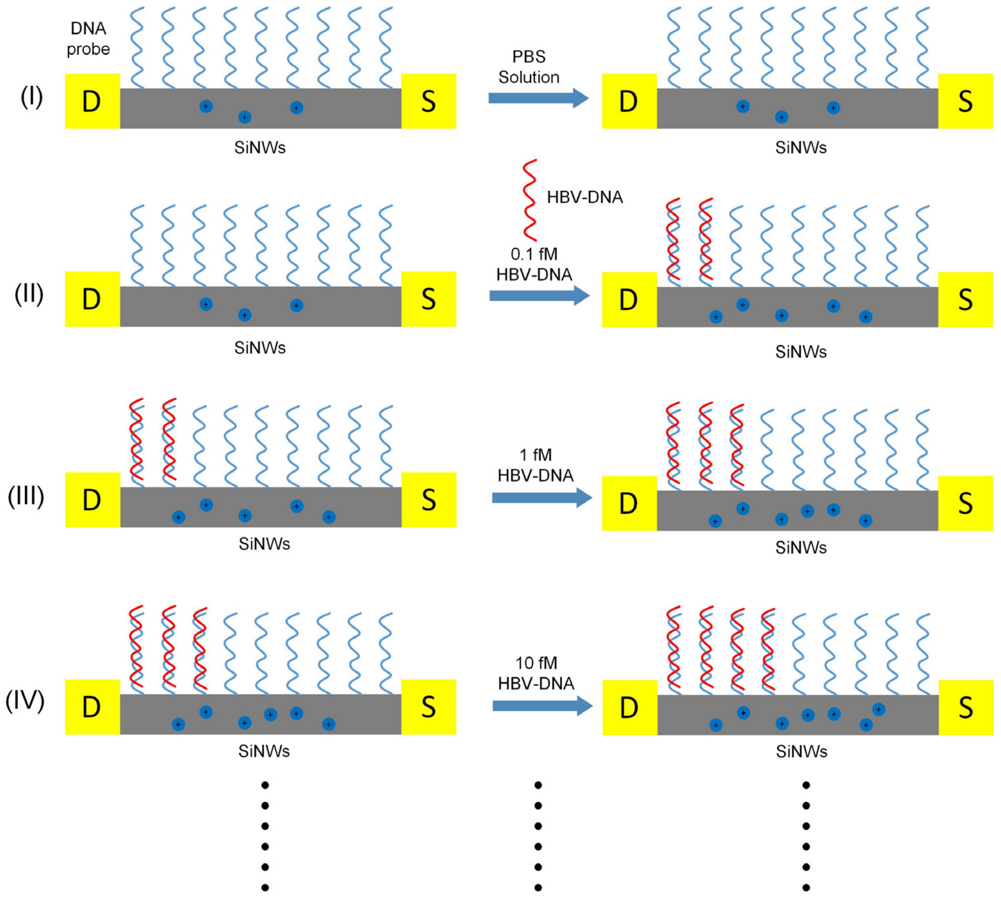

Figure S2. The binding process between probe DNA molecules and HBV-DNA molecules on SiNWs.

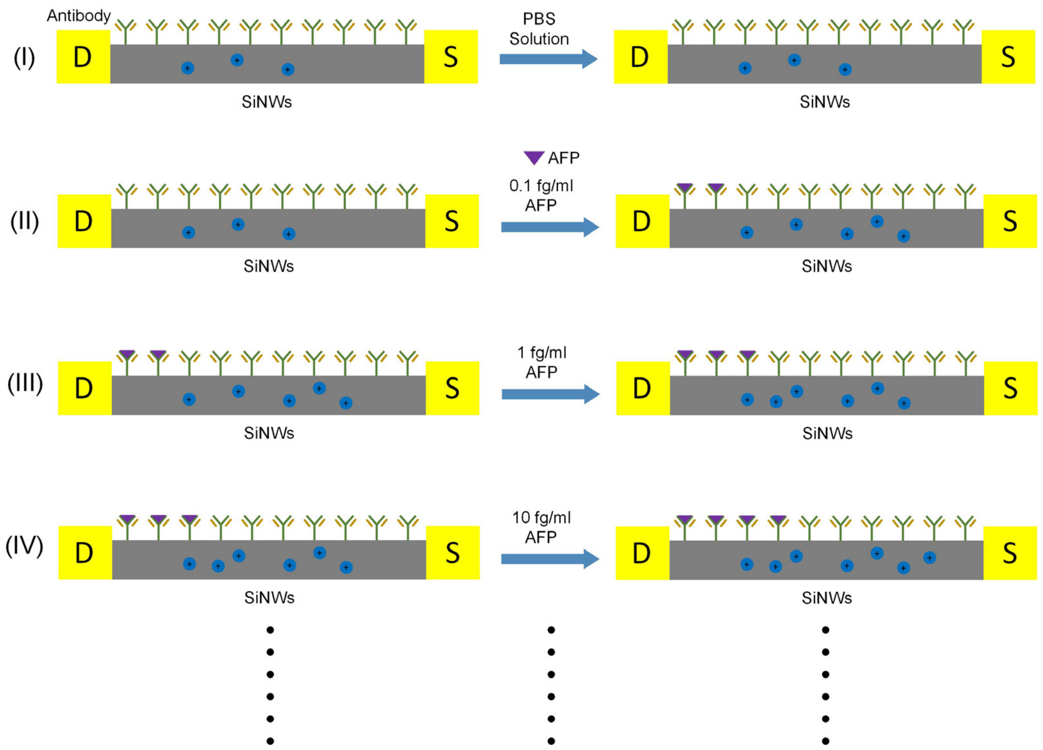

Figure S3. The binding process between antibodies and AFP molecules on SiNWs.
